# Molecular Iodine-Mediated Cyclization of Tethered Heteroatom-Containing Alkenyl or Alkynyl Systems

**DOI:** 10.3390/molecules14124814

**Published:** 2009-11-25

**Authors:** Malose Jack Mphahlele

**Affiliations:** Department of Chemistry, College of Science, Engineering and Technology, University of South Africa, P.O. Box 392, Pretoria 0003, South Africa; E-Mail: mphahmj@unisa.ac.za; Tel.: +27-12-429-8805; Fax: +27-12-429-8549

**Keywords:** iodine, iodocyclization, cyclodehydroiodination

## Abstract

Molecular iodine has established itself as a readily available and easy-to-handle electrophilic and oxidizing reagent used in various organic transformations. In this review attention is focused on the use of molecular iodine in promoting cyclization (iodocyclization and cyclodehydroiodination) of tethered heteroatom-containing alkenyl or alkynyl systems.

## 1. Introduction

In recent years, molecular iodine has received considerable attention as an inexpensive, non-toxic, readily available reagent to effect iodocyclization and cyclodehydroiodination reactions of tethered heteroatom-containing alkenyl or alkynyl systems to afford heterocyclic compounds with many synthetic and biological applications. For example, iodine-promoted cyclization of tethered heteroatom (oxygen-, nitrogen- or sulfur-)-containing alkynes has proven to be an effective method for the synthesis of furans [1,2,3], pyrroles [4,5], thiophenes [6,7] indoles [8,9], benzo[*b*]furans [10,11], and benzo[*b*]thiophenes [12,13,14]. The pyrrole moiety is widely distributed in a large number of naturally occurring compounds which display a variety of physiological properties [15] including antibacterial [16], antiviral [17], and antioxidant activities and also inhibit cytokine-mediated diseases [18,19]. On the other hand, the furan moiety is also found in naturally occurring compounds or synthetic derivatives such as naphtha[2,3-*b*]furan-4,9-diones **1** and naphtha[1,2-*b*]furan-4,5-diones **2** which have been found to exhibit *in vitro* cyctotoxicity against KB cells [20]. Benzo[*b*]thiophene analogues **3a**–**c** prepared through a combination of palladium–mediated coupling and iodine–promoted iodocyclization were found to exhibit tubulin binding activities [6].

**Figure 1:**
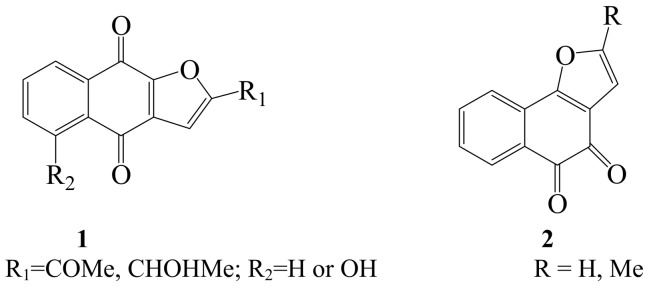
Structures of naphtha[2,3-*b*]furan-4,9-diones **1** and naphtha[1,2-*b*]furan-4,5-diones **2**.

**Figure 2:**
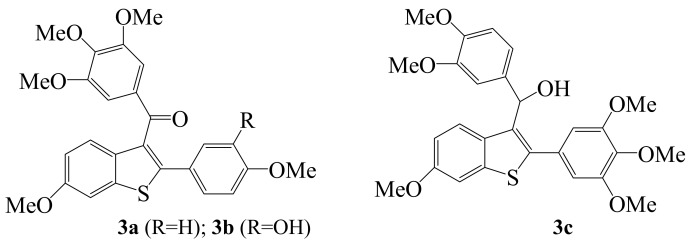
Structures of benzo[*b*]thiophene analogues **3a–c**.

Although the popularity of molecular iodine–mediated cyclization reactions has been increasing over the years, to our knowledge, there is no comprehensive review in the literature on the use of iodine as an electrophile and/or oxidizing agent in the synthesis of heteroatom-containing compounds with potential synthetic and/ or biological applications. Its applications in the oxidation of alcohols and aldehydes to esters, nitriles and amides as well as the introduction of protecting groups and deprotection have however been reviewed in detail before [21]. In another development, Banerjee *et al.* reviewed the application of molecular iodine in esterification, cycloaddition, allyllation of aldehydes, acetalization of carbonyl compounds, acylation of alcohols, synthesis of cyclic ethers and aromatization of α,β-unsaturated ketones [22]. In this review, particular attention is focused on methods that employ molecular iodine as an electrophile and/ or oxidizing agent to promote cyclization of tethered alkenyl and alkynyl derivatives bearing a nucleophilic heteroatom-containing group. 

## 2. Iodine as an Electrophile in Cyclization Reactions

Although halogen molecules on their own are nonpolar, they are easily polarized by the pi electrons of the C=C double bond to become electrophilic. The electrophilic properties of iodine have been exploited over the years to effect cyclization of heteroatom-containing alkenyl and alkynyl derivatives. 

### 2.1. Iodine-promoted cyclization reactions

Halocyclization is a reaction whereby the intramolecular nucleophilic group attacks the carbon–carbon double or triple bond activated by electrophilic halogenating reagent to give cyclic compounds. The outcome of this cyclization strategy which has been exploited in recent years for the synthesis of furans, pyrroles and quinolinones and their analogues is rationalized in terms of the rules previously developed by Baldwin for predicting the relative ease of organic ring-forming reactions [23]. The physical bases for these three rules are the stereochemistry requirements of the transition states for various tetrahedral, trigonal, and digonal systems in nucleophilic, homolytic, and cationic ring closure processes [23]. Iodocyclization of tethered heteroatom-containing alkenyl or alkynyl derivatives as well as iodocyclization of 2-allyl-1,3-dicarbonyl derivatives take advantage of the electrophilic nature of iodine. We herein focus attention on iodine–mediated cyclization reactions involving O-, N- or S-containing group as an intramolecular nucleophile.

#### 2.1.1. Iodocyclization of heteroatom-containing alkenyl derivatives

**Scheme 1:**
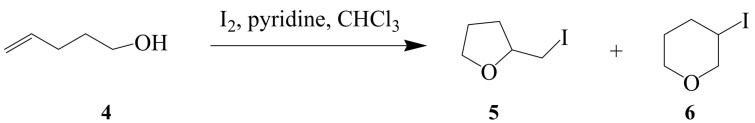
Iodocyclization of 5-penten-1-ol in the presence of pyridine.

**Scheme 2:**
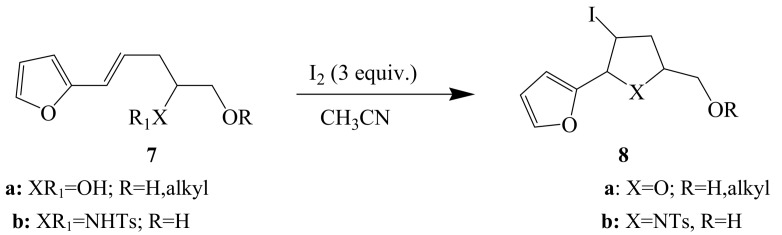
Iodocyclization of furyl-substituted pent-4-ene-1,2-diols.

Iodocyclization of 4-penten-1-ol **4** using iodine in chloroform with 1 equivalent of pyridine is reported to afford mixture of products characterized as the tetrahydrofuran **5** and tetrahydropyran **6** (Scheme 1) [24]. Iodine (3 equiv.) in acetonitrile (CH_3_CN) was found to promote iodocyclization of furyl-substituted pent-4-ene-1,2-diols **7a** (XR_1_=OH; R=H, alkyl) and their sulfonamide derivatives **7b** (XR_1_=NHTs; R=H) to afford iodotetrahydrofurans **8a** (X=O; R=H, alkyl) and 5-furylpyrrolidine-2-methanol **8b** (X=NTs, R=H), respectively (Scheme 2) [4].

**Scheme 3:**
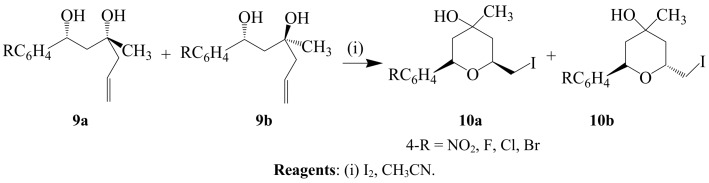
Iodocyclization of alkenyl 1,3-diols.

Tetrahydropyrans **10a** and **b** were prepared in the ratio 2:1 from a diastereomeric mixture of **9a** and **b** using iodine in dry acetonitrile (Scheme 3) [25].

**Scheme 4:**
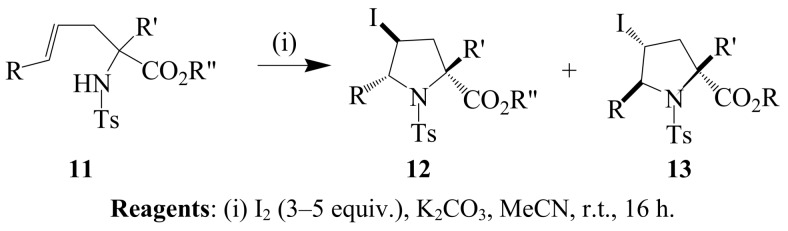
Base-promoted iodocyclization of α-alkenyl-α-amino esters.

Iodocylization of γ-alkenyl-β-enaminoesters and α-alkenyl-β-enaminoesters with iodine-NaHCO_3_ mixture in dichloromethane at room temperature, on the other hand, previously afforded novel 2-, and *N*-substituted 5-methylene-pyrrolidine benzamides and 2-, 3- and *N*-substituted 5-methylene-2-pyrroline benzamides, respectively [26]. Substituted proline derivatives **12** and **13** were prepared in excellent yields through 5-*endo*-iodocylization of the corresponding α-alkenyl-α-amino esters **11** with iodine in the presence and absence of a base (Scheme 4) [27]. The analogous (*E*)-homoallylic sulfonamides have been found to undergo the normally disfavored 5-*endo*-trig iodocyclization in the presence of potassium carbonate or sodium carbonate in acetonitrile to afford *trans*-2,5-disubstituted-3-iodopyrrolidines in high yields [28]. The isomeric *cis*-2,5-disubstituted-3-iodopyrrolidine isomers were isolated as sole products in the absence of a base. It is believed that in the absence of a base, the initial kinetic *trans* products undergo rapid isomerization to afford the thermodynamic *cis*-2,5-disubstituted-3-iodopyrrolidine isomers by a ring opening-ring closure mechanism [28].

**Scheme 5:**
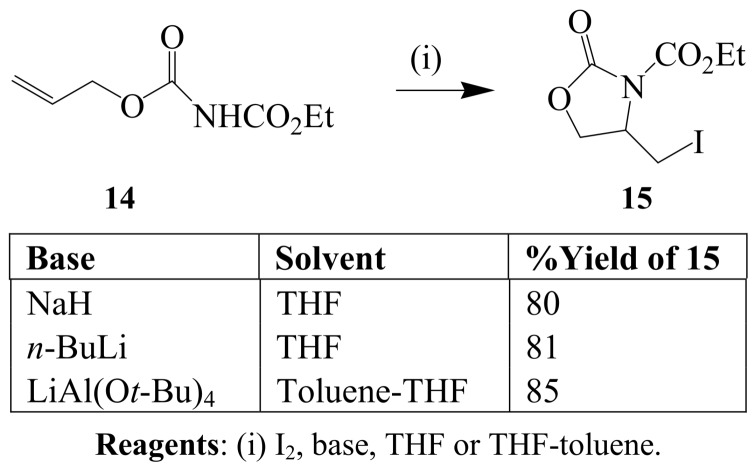
Base-promoted iodocyclization of *N*-ethoxycarbonyl allylcarbamate.

A general method for iodine–mediated cyclization reactions of unsaturated carbamates, ureas and amides which gives *N*-cyclized products as single regio-isomers was achieved in the presence of a strong base such as NaH or LiAl(O*t*-Bu)_4_ [29]. The reaction of *N*-ethoxycarbonyl allylcarbamate **14** with iodine (3 equiv.) in tetrahydrofuran (THF) or toluene-THF mixture in the presence of NaH, nBuLi or LiAl(O*t*-Bu)_4_ afforded the *N*-cyclized product **15** in 58 – 85% yield without traces of the *O*-cyclized derivative (Scheme 5).

**Scheme 6:**
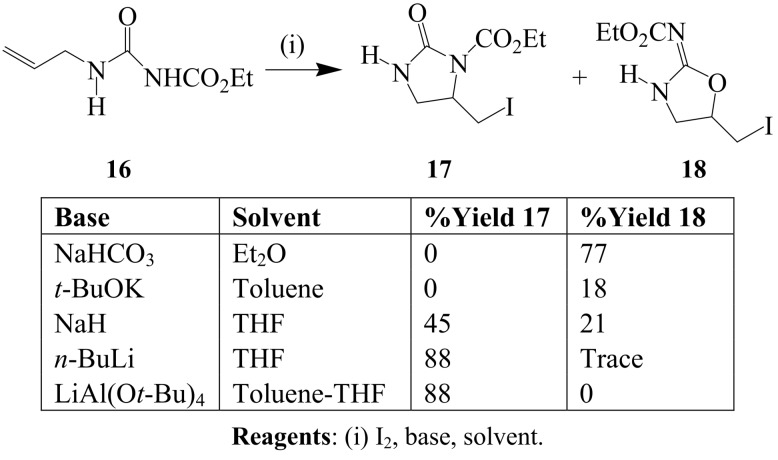
Iodine-mediated cyclization of *N*-ethoxycarbonyl *N’*-allylurea.

Exclusive formation of the *O*-cyclized derivative **18** was observed when *N*-ethoxycarbonyl-*N’*-allylurea **16** was treated with iodine–NaHCO_3_ mixture in ether (Scheme 6) [29]. The *N*-cyclized derivatives of *N*-ethoxycarbonyl *N’*-allylurea **17** were isolated as sole products only when n-BuLi or Li(Al(O*t*-Bu)_4_ were used as bases. This reverse regioselectivity is presumably the result of the tendency for lithium ion to coordinate strongly with oxygen atoms in a six-membered cyclic transition state. Such interaction would render oxygen less nucleophilic and in turn favour nucleophilic attack by nitrogen to afford the *N*-cyclized products.

**Scheme 7:**
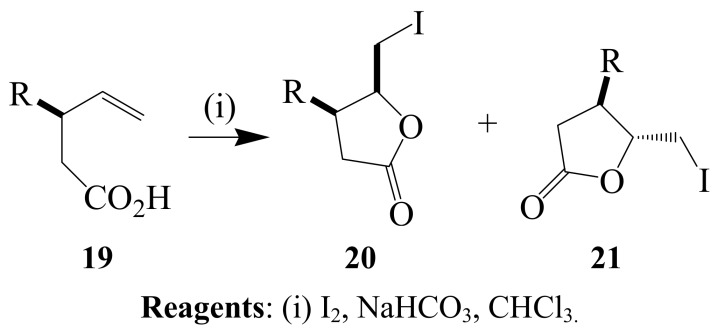
Iodine-promoted cyclization of nonconjugated unsaturated acids.

Ferraz and coworkers previously reported the synthesis of *N*-substituted pyrrole derivatives from alkenyl 1,3-dicarbonyl compounds *via* the formation of iodo-1,3-enamino esters followed by dehydroiodination [30,31]. Iodine-promoted cyclization of **19** to afford mixture of *cis* and *trans* isomers of 4,5-dihydro-5-iodomethyl-4-phenyl-2(3*H*)-furanones **20** and **21** has been described before (Scheme 7) [32]. A detailed review describing examples of iodine–mediated cyclization of nonconjugated unsaturated acids, diallyl-hydroxyacetic acids, benzyl carbamates, norbornene derivatives, aryl-allenoic acids, olefinic amides and γ-methallyl malonic acids to afford iodolactones and lactones was published recently [33].

**Scheme 8:**
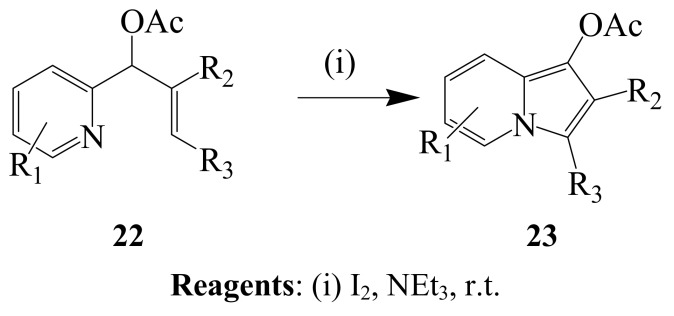
Iodocyclization of (*E*)-(pyridin-2-yl)allyl acetates.

Variously substituted (*E*)-(pyridin-2-yl)allyl acetates **22** were previously subjected to iodine in triethyl amine to afford the corresponding indolizines **23** *via* 5-*endo-trig* iodocyclization (Scheme 8) [34]. In this one-pot reaction sequence, pyridinyl nitrogen was involved as an internal nucleophile of iodocyclization.

**Scheme 9:**
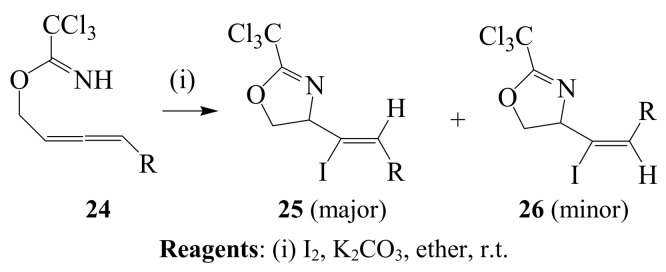
Iodocyclization of the trichloroacetimidate derivatives of primary α-allenic alcohol.

Stereoselective formation of *Z*-4-(1-iodo-2-alkyl)ethylene-2-trichloromethyl-4,5-dihydro-1,3-oxazoles **25** was previously achieved *via* iodocyclization of the trichloroacetimidate derivatives of primary α-allenic alcohol **24** using iodine and potassium carbonate mixture in ether (Scheme 9) [35]. The *Z*-vinyl iodides **25** (R=alkyl) were isolated in high yields (58 – 80%) as major isomers.

#### 2.1.2. Iodocyclization of heteroatom-containing alkynyl and ynone derivatives

**Scheme 10:**
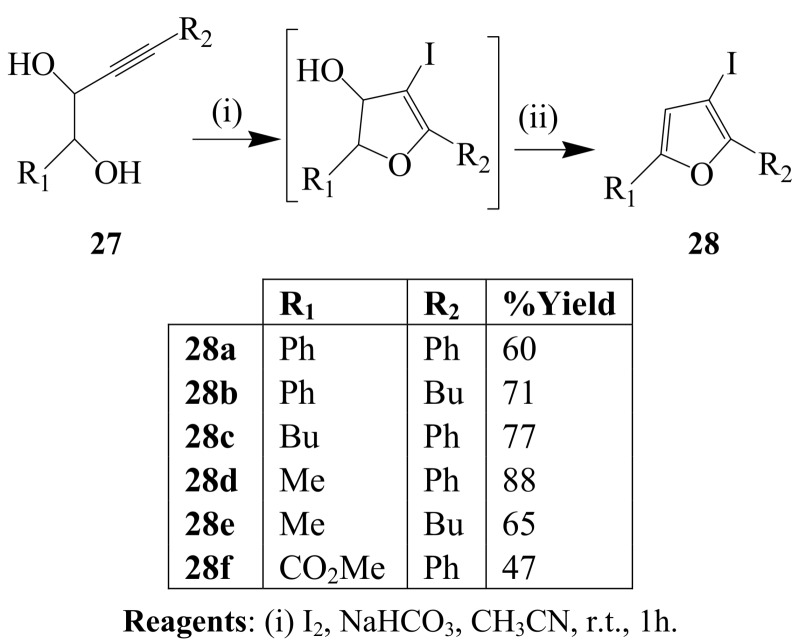
Iodocyclization of 3-alkyne-1,2-diols.

Iodine and sodium bicarbonate mixture in dichloromethane at 0 ºC was found to promote a one-pot 5*-endo-dig* cyclization of 3-alkynyl-1,2-diols and subsequent dehydration to afford β-iodofurans in high yield [1,3]. In a follow up investigation, Knight and coworkers treated a series of 3-alkyne-1,2-diols **27** with iodine (3.3 equiv.) and NaHCO_3_ (3.3 equiv.) mixture in MeCN at room temperature and isolated the corresponding β-iodofurans **28** (Scheme 10) [36].

**Scheme 11:**
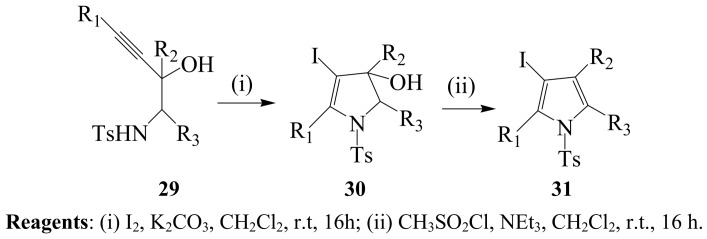
Iodocyclization of homopropargylic sulfonamides.

A representative series of homopropargylic sulfonamides have been found to undergo 5-*endo*-*dig* cyclization upon exposure to excess iodine in acetonitrile in the presence of potassium carbonate to afford 4-iodo-2,3-dihydropyrroles and β-iodopyrroles substituted with ester group at the 2-position [4]. A range of 3-hydroxy-2-sulfonylamino-4-alkynes **29** was also treated with iodine–K_2_CO_3_ mixture in dichloromethane to afford systems **30**, which were in turn dehydrated using methanesulfonyl chloride in dichloromethane in the presence of triethylamine to yield iodopyrroles **31** (Scheme 11) [37].

**Scheme 12:**
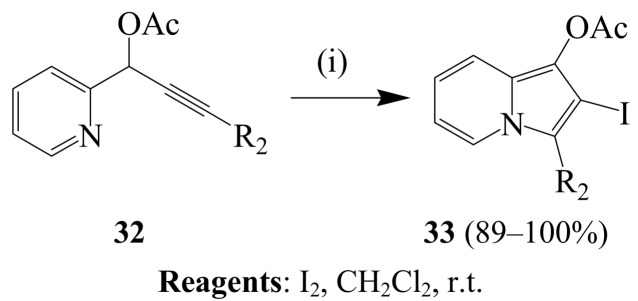
Iodocyclization of (*E*)-(pyridin-2-yl)alkynyl acetates.

**Scheme 13:**
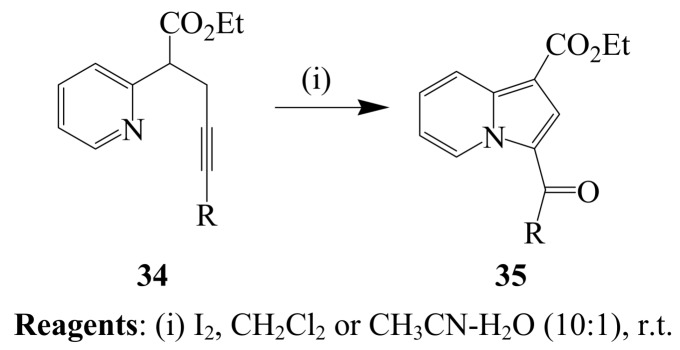
Iodocyclization of 2-pyridin-2-yl-pent-4-ynoic acid ethyl esters.

A pyridinyl nitrogen was involved as an internal nucleophile during iodine-mediated 5-*endo-dig* cyclization of series of propargylic acid esters **32** in dichloromethane at room temperature to afford the corresponding highly functionalized indolizines **33** (R_2_=alkyl, aromatic or heteroaromatic) in high yields (Scheme 12) [38]. Under similar reaction conditions, the analogues 2-pyridin-2-yl-pent-4-ynoic acid ethyl esters **34** afforded the corresponding 3-acylated indolizines **35** (R=alkyl or aryl) *via* iodine-mediated hydrative cyclization (Scheme 13) [39]. Improved yields were observed when acetonitrile-water mixture (10:1, v/v) was used as solvent. The mechanism of this transformation is believed to involve 5-*exo-dig* iodocyclization, deprotonation, incorporation of another iodo group, deprotonation and subsequent replacement of the diiodo group by water.

**Scheme 14:**
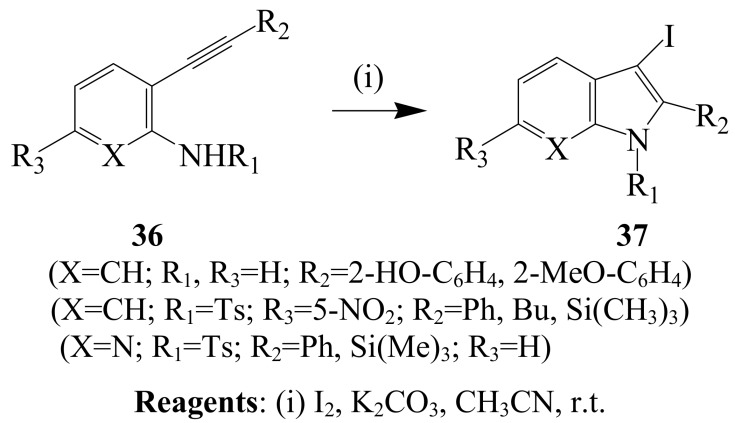
Iodine-promoted cyclization of 2-alkynylanilines, *N*-tosyl-2-alkynylanilines and 2-(*N*-tosyl)-3-alkynylpyridine derivatives.

Iodine–promoted cyclization of 2-alkynylaniline **36** (X=CH; R_1_, R_3_=H; R_2_=2-HO-C_6_H_4_, 2-MeO-C_6_H_4_) afforded iodoindoles **37** (X=CH; R_1_, R_3_=H; R_2_=2-HO-C_6_H_4_, 2-MeO-C_6_H_4_) in low yields (10–20%) presumably due to the presence of free hydroxyl or amino groups on the substrate (Scheme 14) [40]. Treatment of the analogous *N*-tosyl-2-alkynylaniline derivatives **36** (X=CH; R_1_=Ts; R_2_=Ph, Bu, Si(CH_3_)_3_; R_3_=5-NO_2_) and 2-(*N*-tosyl)-3-alkynylpyridines (X=N; R_1_=Ts; R_2_=Ph, Si(CH_3_)_3_; R_3_=H) with iodine (3 equiv.) and K_2_CO_3_ (3 equiv) in acetonitrile at 0–20 °C afforded the corresponding iodoindoles (82–95%) and azaindoles (75–89%), respectively [9]. Iodocyclization of 2-alkynyldimethylaniline derivatives (R_1_=Me; R_2_=alkyl) previously afforded 3-iodoindole (R_2_=Me) [41]. Electrophilic cyclization of *N,N*-dialkyl-*o*-(alkynyl)anilines with iodine in dichloromethane, on the other hand, led to the isolation of *N*-alkyl-3-iodoindoles in excellent yields [8].

**Scheme 15:**
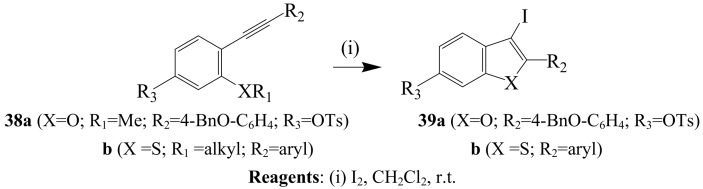
Iodine-promoted cyclization of 2-alkynylanisoles and alkyl(2-alkynylphenyl) sulfides.

The 5-*endo*-dig-iodocyclization of 2-alkynylphenols with iodine in the presence of NaHCO_3_ at room temperature produced 2-substituted 3-iodobenzo[*b*]furans, which are useful synthetic intermediates for the preparation of 2,3-disubstituted benzo[*b*]furans *via* Pd-catalyzed reactions [13,14,42]. Iodocyclization of 2-alkynylanisole derivatives **38a** and the alkyl(2-alkynylphenyl) sulfides **38b** afforded the corresponding iodofuran **39a** [40] and 3-iodobenzo[*b*]thiophenes **39b** [41], respectively (Scheme 15). *o-*(Phenylethynyl)thioanisole and *o*-(1-alkynyl)thioanisoles have also been treated with iodine (1.5 equiv.) in dichloromethane at room temperature to afford 3-iodo-2-(alkyl/aryl)benzo[*b*]thiophenes in more than 95% yield [13]. Flynn and coworkers previously employed this strategy in the synthesis of novel tubulin polymerization inhibitors **3** from the corresponding 3-iodobenzo[*b*]thiophene prepared, in turn, *via* 5-*endo-dig* iodocyclization of benzyl *o*-ethynylphenyl sulfides with iodine in dichloromenthane [12].

**Scheme 16:**
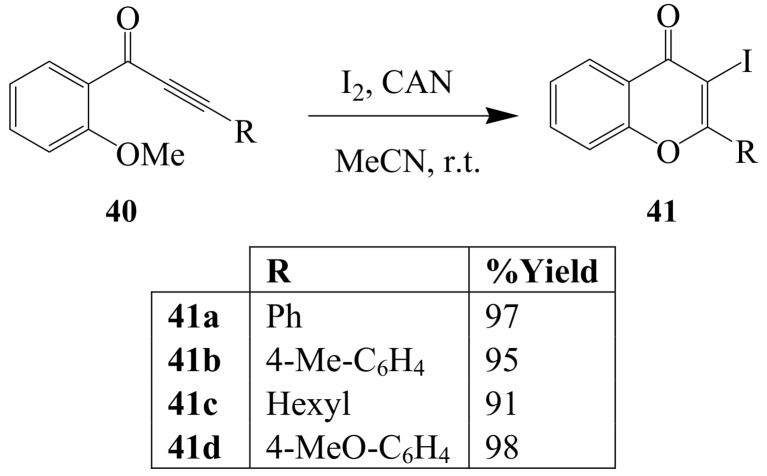
Iodine-CAN mediated cyclizations of (2-methoxyaryl) alk-2-yn-1-ones.

**Scheme 17:**
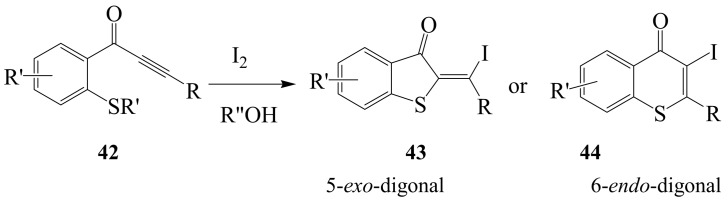
Iodine-mediated cyclization of 2-(*N,N*-dimethylaminophenyl)alk-2-yn-1-ones.

Iodine–cerium(IV) ammonium nitrate (CAN) mixture in acetonitrile at room temperature previously induced cyclization of (2-methoxyaryl)-substituted ynones **40** to produce 3**-**iodochromenones (3-iodo-4*H*-1-benzopyran-4**-**ones) **41** in excellent yields (Scheme 16) [43]. In another development, the phenyl derivative **40** (R=Ph) was transformed to **41a** using iodine in dichloromethane at room temperature [40]. 1-(2-Alkylthiophenyl)alk-2-yn-1-ones **42** and their 1-(2-alkylthiophenyl)alk-2-yn-1-ol derivatives exhibited strong bias toward the 5-*exo*-dig pathway to give **43** instead of the 6-*endo*-dig pathway leading to 3-iodothioflavones **44** (Scheme 17) [14,41]. 

**Scheme 18:**
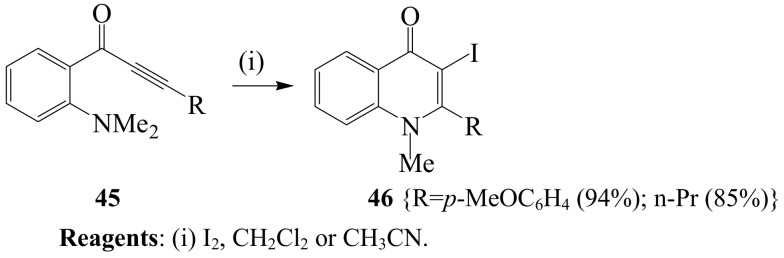
Iodine-mediated cyclization of 2-(*N,N*-dimethylamino) substituted ynones.

Highly selective 5-*exo*- and 6-*endo*-*dig* iodocyclization protocols that give direct access to a variety of indoles and quinolines have been described in literature [41]. Iodocyclization of the dimethylamino systems **45** using iodine in dichloromethane or acetonitrile proved highly selective for the 6-*endo*-digonal pathway to afford 2-substituted 3-iodo-1-methylquinolin-4(1*H*)-ones in high yield **46** (Scheme 18) [41].

**Scheme 19:**
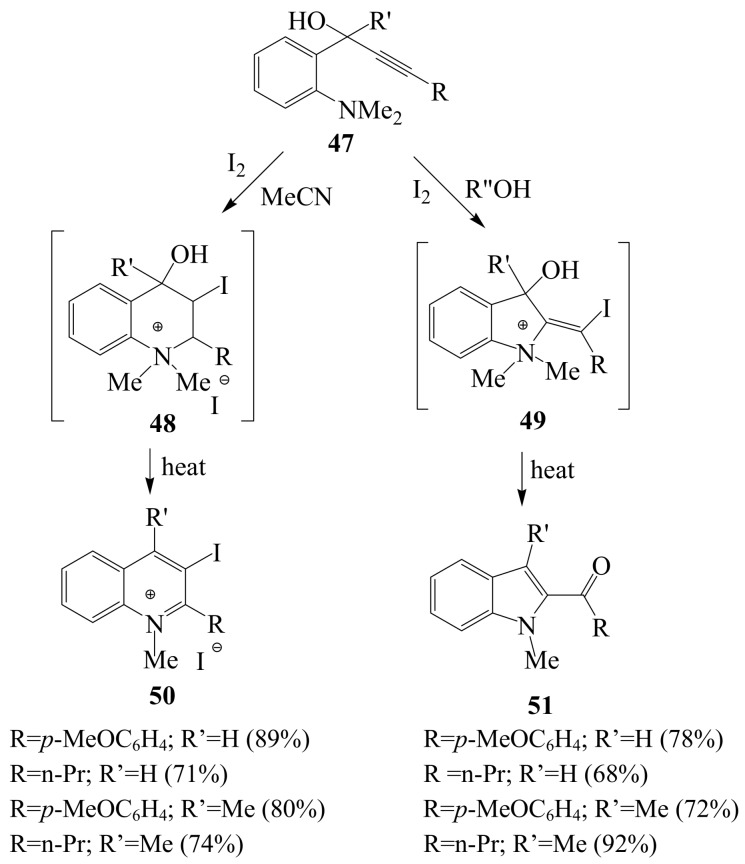
Iodine-mediated cyclization of 2-(*N,N*-dimethylaminophenyl)alk-2-yn-1-ols in polar aprotic (CH_3_CN) and polar protic (methanol or ethanol) solvents.

When secondary and tertiary alcohols **47** were subjected to iodine in acetonitrile or dichloromethane, the *endo* product **48** was produced exclusively and then transformed to quinolium salt **50** by direct heating of the reaction mixture (Scheme 19) [41]. The 5-*exo*-*dig* products **49** which were isolated as 2-acylindoles **51** were found to form exclusively in protic solvents such as methanol or ethanol. This strategy was previously applied for the synthesis of novel tubulin polymerization inhibitors **3a-c** from the corresponding 3-iodobenzo[*b*]thiophene prepared, in turn, *via* 5-*endo-dig* iodocyclization of benzyl *o*-ethynylphenyl sulfides with iodine in dichloromenthane [6,14].

**Scheme 20:**
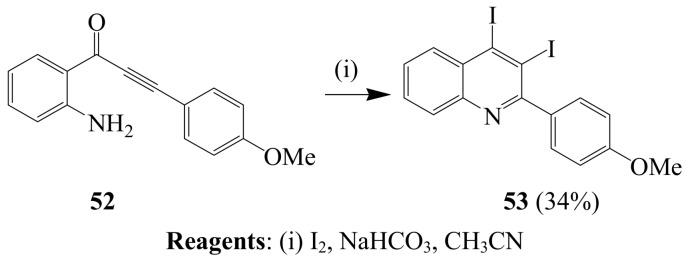
Iodine-NaHCO_3_ promoted cyclization of β-(2-aminophenyl)-α,β-ynone.

The reaction of β-(2-aminophenyl)-α,β-ynone **52** with I_2_ and NaHCO_3_ in CH_3_CN afforded the 3,4-diiodo-2-(4-methoxyphenyl)quinoline **53** in 34% yield (Scheme 20) [44]. This observation was found to be remarkably different from the regio-controlled iodoaminocyclization reaction of related derivatives.

**Scheme 21:**
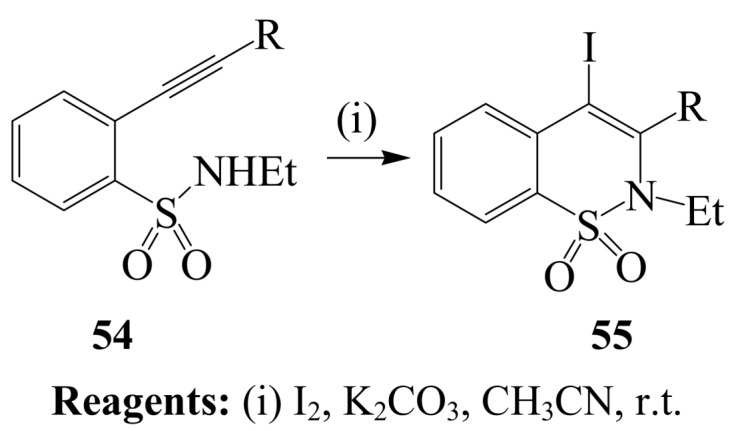
Iodine-K_2_CO_3_ mediated cylization of *o*-(1-alkynyl)benzenesulfonamides.

Molecular iodine in acetonitrile effected regioselective iodocyclization of *o*-(1-alkynyl)benzenesulfonamides **54** to yield a variety of 4-iodo-2*H*-benzo[*e*][1,2]thiazene-1,1-dioxides **55** (Scheme 21) [45]. The iodocyclization step was found to tolerate a variety of functional groups such as hydroxyl, chloro, cyano, and methoxy substituent to produce a six-membered ring exclusively.

**Scheme 22:**
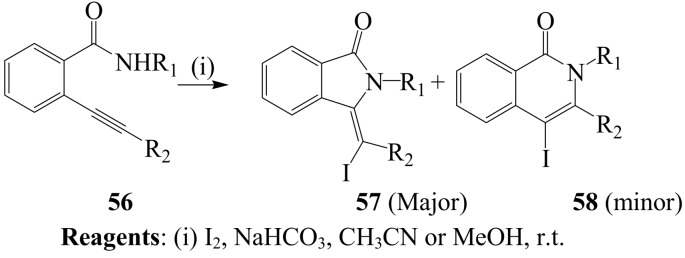
Iodine-NaHCO_3_ mediated cylization of *o*-(1-alkynyl)benzamides.

A series of *o-*(1-alkynyl)benzamides **56** were previously treated with iodine in dichloromethane at room temperature to afford the corresponding isoindolin-1-ones **57** and isoquinolin-1(2*H*)-ones **58** as a mixture (Scheme 22) [46]. Better regioselectivity was observed for iodine compared to other electrophiles (ICl, NBS, PhSeCl and *p*-NO_2_C_6_H_4_SCl) and this improved in acetonitrile or methanol in the presence of a base.

#### 2.1.3. Iodocyclization of *ortho*-allylphenols

**Scheme 23:**
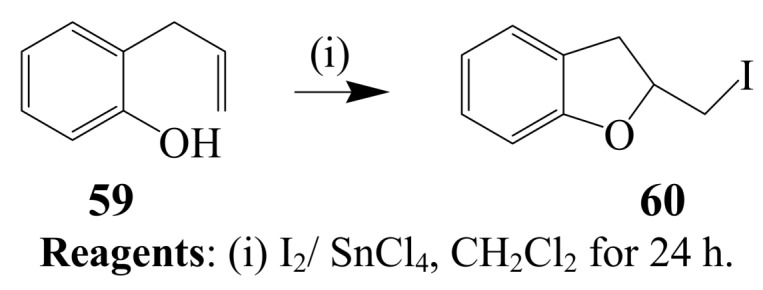
Iodine-SnCl_4_ mediated cylization of 2-allylphenol.

**Scheme 24:**
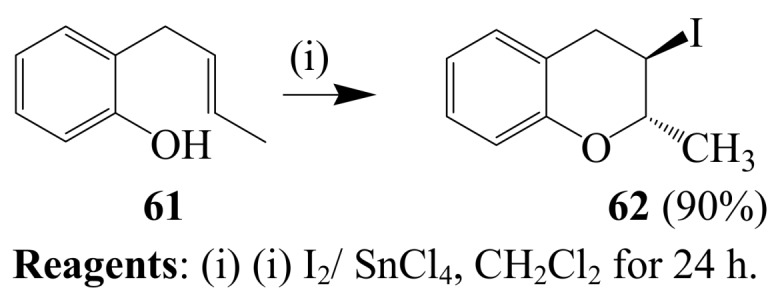
Iodine-SnCl_4_ mediated cylization of 2-(2-butenyl)phenol.

Iodoenolcyclization of *ortho*-allylphenol **59** using either I_2_-SnCl_4_ mixture in dichloromethane [47] or I_2_ and Ethopropazine, EPZ-10 (a clay-supported ZnCl_2_ catalyst) mixture or ZnCl_2_ in methanol is reported to afford the 2-iodomethyl-2,3-dihydrobenzofuran **60** (Scheme 23) [48]. Iodoenolcyclization of 2-(2-butenyl)phenol **61** with I_2_-SnCl_4_ mixture in dichloromethane at room temperature, on the other hand, previously afforded 3-iodo-2-methylbenzopyran **62** in excellent yield (Scheme 24) [47]. Muzart *et al*. [49], reported the reaction of a variety of 2-allylphenols with iodine in water which led to the corresponding 2-iodomethyl-2,3-dihydrobenzofurans in the absence of any additives or organic solvents. 

#### 2.1.4. Iodocyclization of 2-allyl-1,3-dicarbonyl derivatives 

**Scheme 25:**
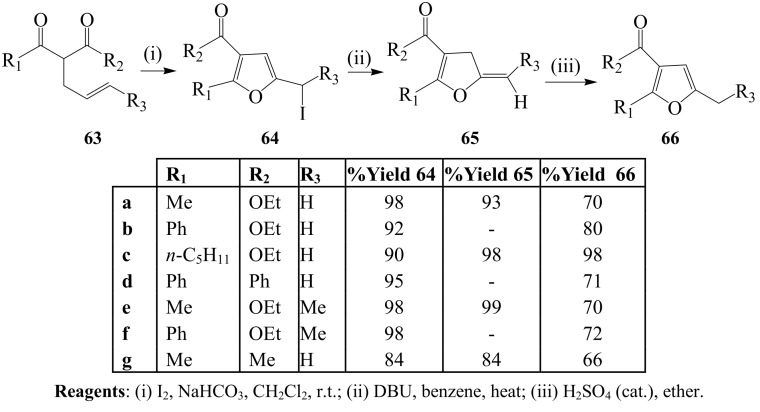
I_2_–NaHCO_3_ mediated iodoenolcyclization of 2-alkenyl-1,3-dicarbonyl compounds.

A convenient approach to furan derivatives by iodine-induced cyclization of 2-alkenyl substituted 1,3-dicarbonyl compounds was first reported by Antonioletti and coworkers [50]. These authors subjected a series of 2-alkenyl substituted 1,3-dicarbonyl compounds **63** to I_2_–NaHCO_3_ mixture in dichloromethane at room temperature to afford 5-iodoalkyl-4,5-dihydrofurans **64** (Scheme 25). Treatment of the latter with 1,8-diazobicyclo[5.4.0]undec-7-ene (DBU) in refluxing benzene afforded 5-alkylidene-4,5-diydrofurans **65**, which were in turn isomerized to the corresponding 2,3,5-trisubstituted furans **66** in ether using an acid catalyst. The generality of this reaction was demonstrated in a follow up study involving treatment of 2-alkenyl-1,3-dicarbonyl derivatives under similar reaction conditions to yield series of 2,3,4,5-tetrasubstituted furans [51].

**Scheme 26:**
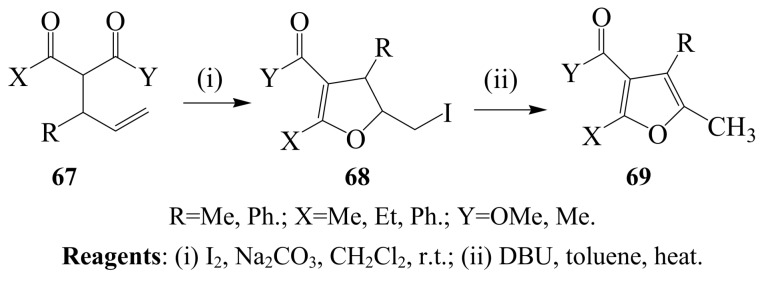
I_2_–Na_2_CO_3_ mediated iodoenolcyclization of 2-allyl-1,3-dicarbonyl derivatives.

Antonioletti’s group also showed that I_2_–Na_2_CO_3_ mixture promotes iodoenolcyclization of 2-allyl-1,3-dicarbonyl derivatives **67** bearing mono and disubstituted double bonds in dichloromethane at room temperature to afford diastereomeric mixtures of 5-iodomethyl-4,5-dihydrofuran derivatives **68** which can be dehydroiodinated using DBU to afford **69** (Scheme 26) [52,53]. Alkyl substituents were found to favour *trans* 5-iodomethyl-4,5-dihydrofuran isomers, whereas aromatic substituents led to *cis* isomers.

**Scheme 27:**
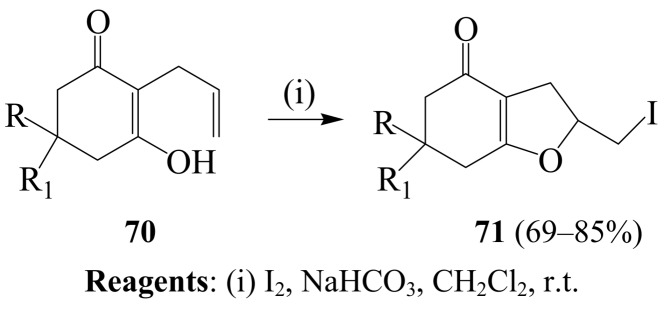
Iodine-mediated cylization of 2-allylcyclohexane-1,3-diones.

The 2-iodomethyl-3,5,6,7-tetrahydrobenzofuran-4-ones have also been recently prepared by polymer-supported selenium-induced electrophilic cyclization of allyl substituted 1,3-dicarbonyl compounds followed by cleavage of the selenium linkers using CH_3_I/NaI in DMF [24]. In another development, Ferraz and coworkers applied I_2_–NaHCO_3_ mixture in dichloromethane at room temperature to series of α-alkenyl β-keto esters and γ-alkenyl β-keto esters bearing mono or disubstituted double bond to afford variously substituted iodocyclic ethers [54] Among the systems employed as substrates were the 2-allyl-1,3-cyclohexanedione derivatives **70**, which afforded 2-iodomethyl-3,5,6,7-tetrahydrobenzofuran-4-ones **71** in high yield (Scheme 27) [54].

**Scheme 28:**
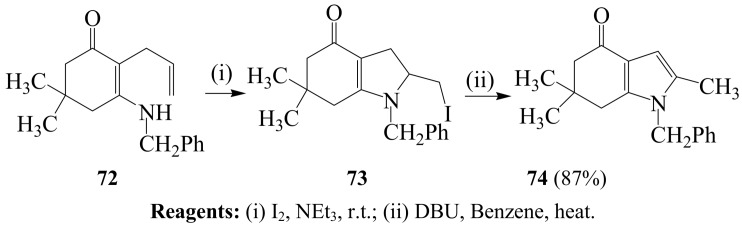
Iodocyclization of 2-allyl-3-benzylamino-5,5-dimethyl-2-cyclohexen-1-one.

Ferraz and coworkers also subjected 2-allyl-β-benzylaminodimedone **72** to I_2_–NEt_3_ mixture to afford 2-iodomethyl-6,6-dimethyl-1-(phenylmethyl)indol-4-one **73** followed by its dehydro-halogenation with 1,8-diazobicyclo[5.4.0]undec-7-ene (DBU) to form 4-oxo-6,7-dihydroindole **74** in 87% yield (Scheme 28) [30]. However, these authors did not provide the corresponding analytical data and the yield for compound **73**, which is implicated in the reaction and the generality of this reaction has not been demonstrated.

**Scheme 29:**
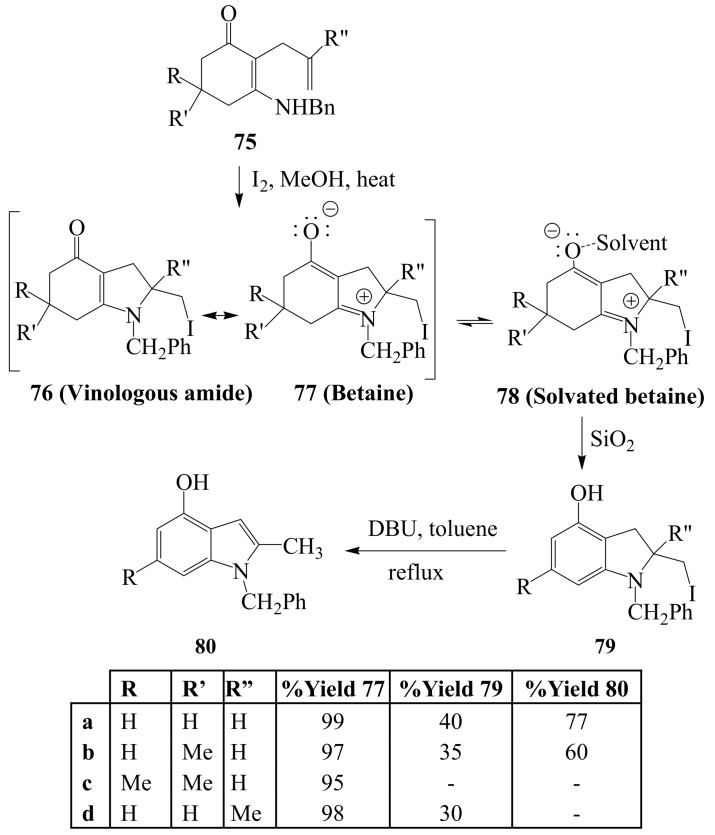
Iodine-methanol promoted cyclization of 2-allyl-3-benzylamine-2-cyclohexen-1-ones.

A series of 2-allyl-3-benzylamino-2-cyclohexenones **75** were recently subjected to iodine–methanol mixture under reflux to afford products characterized by combination of NMR (^1^H- and ^13^C-), IR and mass spectroscopic techniques as the conjugated iodolium betaine derivatives of 2-iodomethyltetrahydroindolones **77** (Scheme 29) [55]. The zwitterionic nature of the products in solution and in the solid state was also confirmed by their chemical behavior and the experimental data were corroborated by information from quantum chemical calculations. Several attempts to dehydrohalogenate systems **77** in analogy with strategy previously employed by Ferraz and coworkers [31] on product **73** above led to complicated mixtures of products. Compounds **79a**, **b** and **d**, however, aromatized on attempted purification on silica gel column to afford the corresponding 4-hydroxy-2-iodomethyldihydroindole derivatives **80** in low yields due to decomposition. The observed stability of these conjugated iodolium betaine derivatives is attributed to the increased propensity of nitrogen for electron pair delocalization resulting in a strong C_2P_–N_2P_ pi bond interaction.

**Scheme 30:**
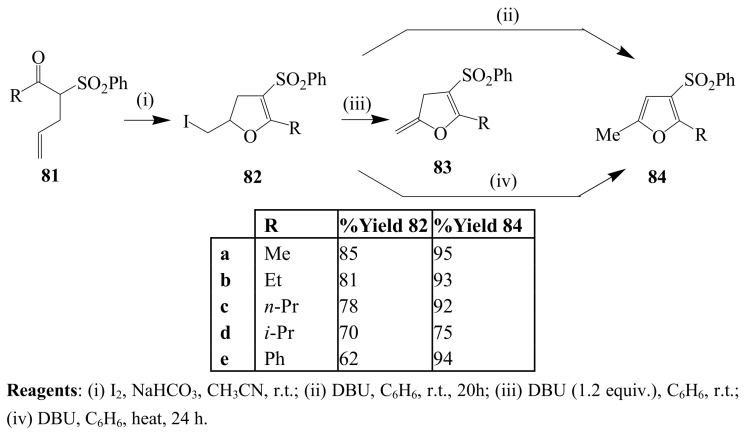
Iodocyclization of β-keto sulfones and subsequent dehydroiodination.

Lee and Oh previously subjected α-allyl substituted β-keto sulfones **81** to I_2_–NaHCO_3_ mixture in acetonitrile and isolated the corresponding 4,5-dihydro-5-iodomethylfurans **82** (Scheme 30) [56]. Dehydroiodination with DBU (1.2 equiv.) in benzene at room temperature afforded 4,5-dihydro-5-methylenefuran **83**. Direct one-pot dehydroiodination–isomerization to furan derivatives **84** was achieved through the use of excess DBU (3–5 equiv.) in benzene at room temperature or under reflux.

**Scheme 31:**
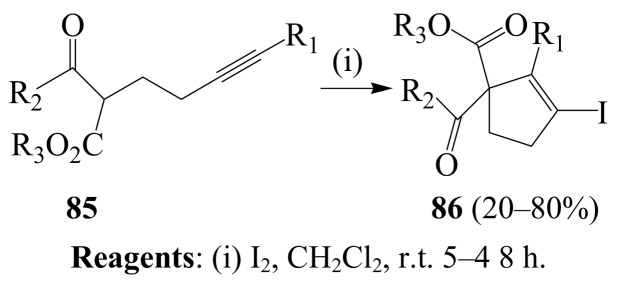
Iodine-mediated carbocyclization of active methylene compounds.

In another development involving iodine–mediated cyclization, a series of δ-alkynyl-β-ketoesters **85** were reacted with iodine in dichloromethane at room temperature for several hours (Scheme 31) [57]. This 5-*endo-dig* mode of carbocyclization of active methylene compounds **85** onto terminal and internal alkynes led to novel iodocyclopentenes **86** in 20–80% yield.

## 3. Combined Electrophilic and Oxidative Properties of Iodine in Cyclization Reactions

Although the combined electrophilic and oxidizing properties of iodine have been exploited in the synthesis of heteroatom-containing cyclic compounds, such reactions do not feature at all in the recent reviews on the application of molecular iodine in organic transformation [21,22].

### 3.1. Iodine-mediated oxidative cyclization reactions

**Scheme 32:**
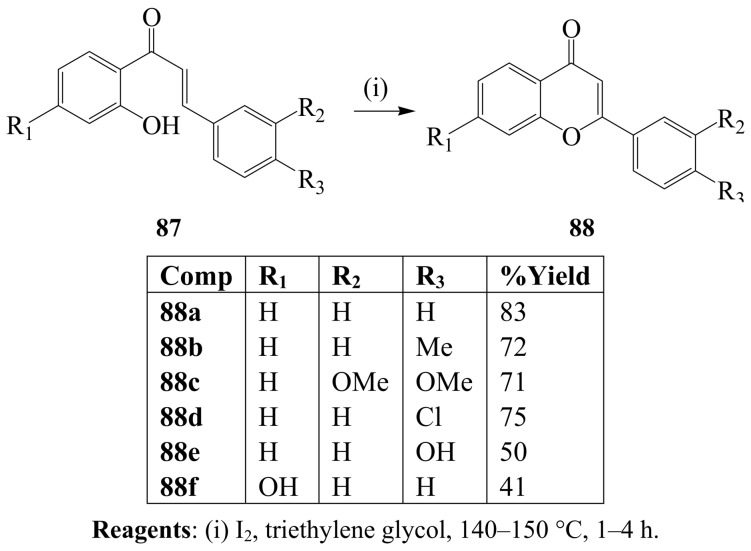
One-pot iodocyclization and oxidative dehydrogenation of 2-hydroxy-3-phenylprop-2-en-1-ones.

Iodine in refluxing triethylene glycol previously promoted oxidative cyclization of 1,3-diphenyl-prop-2-en-1-ones **87** to afford the A- and B-ring substituted flavones **88** (Scheme 32) [58]. The mechanism of this reaction is believed to involve initial electrophilic addition of iodine to the double bond followed by β-elimination of HI. Conjugate addition of the hydroxyl group then affords the 3-iodo flavanone derivative which in turn undergoes β-elimination of HI to afford the flavone. A one-pot iodine–mediated cyclization and oxidative dehydrogenation of 2-hydroxy-3-(4’-methylsulfonyl-phenyl)prop-2-en-1-ones in refluxing dimethylsulfoxide (DMSO) previously afforded 2-{4’-(methyl-sulfonyl)phenyl}benzopyran-4-one as a precursor for the synthesis of 2,3-diarylbenzopyran derivatives with potential to serve as cyclooxygenase-2(COX-2) inhibitors [60]. Use of iodine in triethylene glycol or DMSO has been found to be superior to cyclodehydrogenation of 2-hydroxychalcones with 2,3-dichloro-5,6-dicyano-*p*-benzoquinone (DDQ) in refluxing dioxane, which leads to mixtures of flavanones (3–13%), flavones (28–42%) and aurones (3–17%) [60].

**Scheme 33:**
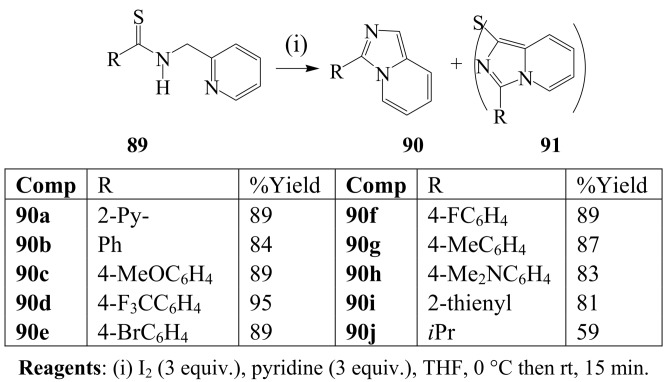
Iodine-mediated oxidative desulfurization promoted cyclization of thioamides.

Iodine-pyridine mixture in THF previously effected one-pot oxidative desulfurization and cyclization of *N-*2-pyridylmethyl thioamides **89** to afford 2-azaindolizines (imidazo[1,5-*a*]pyridines **90** (59 – 95%) and sulfur-bridged 2-azaindolizine dimer **91** (*ca*. 7%) (Scheme 33) [61]. Prolonged reaction time (21 hours) in THF or DMF however led to mixtures of **90** and **91** in comparable yields depending on the nature of substituent on the R group.

### 3.2. Iodine-mediated cyclization and oxidative aromatization reactions

**Scheme 34:**
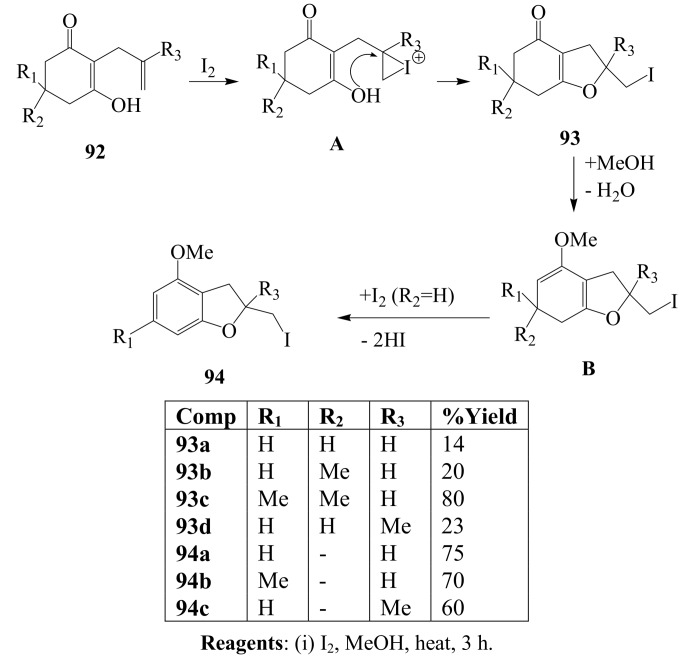
One-pot iodocyclization and oxidative aromatization of 2-allylcyclohexenones.

Progress in modern synthesis is dependent on development of novel methodologies and the combined electrophilic and oxidative properties of iodine were exploited further to synthesize novel iodofunctionalized heterocyclic compounds in a one-pot operation. The strategy involved treatment of 2-allylcyclohexenone derivatives **92** with iodine in refluxing methanol to afford a mixture of 2-iodomethyl-3,5,6,7-tetrahydrobenzofurans **93** (minor) and 2-iodomethyl-4-methoxy-2,3-dihydrobenzo-furans **94** (major) (Scheme 34) [62]. Products **93** are the result of the *exo*-trig type of cyclization which is more favoured than *endo*-trig type of cyclization according to Baldwin’s rule [23]. On the other hand, the formation of products **94** was interpreted as a consequence of an initial 1,2-addition of methanol to **93** followed by dehydration and oxidative aromatization. The use of iodine-methanol mixture as an oxidant to effect aromatization of cyclohexenones to anisole derivatives was first reported in 1980 by Tamura and Yoshimoto [63]. The generality of this aromatization reaction was later demonstrated by several researchers who employed this mixture on cyclohexenone derivatives [64,65,66,67,68,69,70,71,72] and their heterocyclic analogues [73] to prepare novel aromatic compounds that would be difficult to synthesize otherwise.

**Scheme 35:**
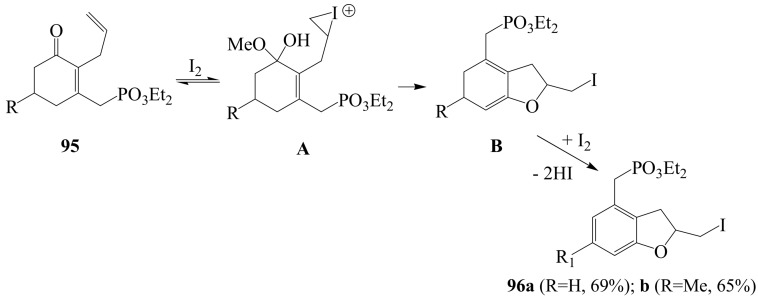
Iodocyclization and oxidative aromatization of diethyl {[2-(2-propenyl)cyclohexenone]methyl}phosphonates.

Under similar reaction conditions applied to systems **92**, diethyl {[2-(2-propenyl)cyclo-hexenone]methyl}phosphonates **95** afforded the corresponding 4-[(diethoxyphosphonyl)methyl]-2-iodomethyl-2,3-dihydrobenzofuran derivatives **96a** and **b** as sole products (Scheme 35) [62]. The observed result was interpreted as a consequence of initial formation of hemiacetal **A** from **95** followed by cyclization and the loss of methanol to form **B**. Iodine-assisted dehydrogenation of **B** would then result in the formation of **96**.

Iodine-methanol reaction mixture has established itself to be more effective than metal–catalyzed aromatization of substituted cyclohexenones to the corresponding phenols or phenol ethers [74,75,76,77,78]. This reagent mixture was also found to be superior to the use of DDQ in dioxane, which was previously employed to dehydrogenate 5-acetyl-4-oxo-4,5,6,7-tetrahydrobenzofuran and methyl-4-oxo-4,5,6,7-tetrahydrobenzofuran-5-carboxylate [78].

## 4. Conclusions

Iodine has established itself as an efficient, readily available and easy-to-handle electrophilic reagent to effect halocyclization reactions to afford novel iodofunctionalized heterocyclic molecules that serve as versatile intermediates in synthetic organic chemistry [79]. Carbon-heteroatom bond-forming reactions constitute the central theme of organic synthesis, and progress in modern synthesis is dependent on development of novel methodologies for the same. Series of 3-iodoindoles prepared *via* iodocyclization of the corresponding *N,N*-dialkyl-*o*-(1-alkynyl)anilines, for example, were recently subjected to palladium–catalyzed Sonogashira and Suzuki cross coupling reactions in solution and on a solid support to afford a 42-member library of 1,2,3,5-tetrasubstituted indoles after cleavage from the support [80]. In summary, molecular iodine has allowed in the last years a great advance in organic chemistry in the synthesis of heterocyclic compounds with many applications. Moreover, the combined electrophilic and oxidative potential of iodine can be exploited to synthesize novel aromatic and heteroaromatic compounds that would be difficult to synthesize otherwise. 
